# Supplementary material for: MetaRibo-Seq measures translation in microbiomes
Source: Nat Commun. 2020 Jun 29;11:3268. doi: 10.1038/s41467-020-17081-z (PMC7324362; doi:10.1038/s41467-020-17081-z)
Supplement: Supplementary file 10 — Supplementary Data 7 [file 41467_2020_17081_MOESM10_ESM.zip › File2/Confidence_High_Taxonomy/319497_out.krona.html]

Javascript must be enabled to view this page.

members
magnitude
magnitudeUnassigned
count
unassigned
taxon
rank

319497\_out

72

72
2
superkingdom

phylum
72
1239

72
186801
class

order
72
186802

72
186803
family

841
72
genus

1
2292367
species

SRS015579\_contig\_number\_25852


SRS011084\_contig\_number\_23658SRS013158\_contig\_number\_1766SRS014287\_contig\_number\_15849SRS014613\_contig\_number\_20850SRS014855\_contig\_number\_14677SRS014923\_contig\_number\_41389SRS015065\_contig\_number\_25386SRS015190\_contig\_number\_contig-100\_1204.95507SRS015264\_contig\_number\_21766SRS015431\_contig\_number\_2334SRS015578\_contig\_number\_27029SRS015854\_contig\_number\_17999SRS015890\_contig\_number\_15752SRS015960\_contig\_number\_8749SRS016267\_contig\_number\_192SRS016335\_contig\_number\_21223SRS017821\_contig\_number\_22703SRS018984\_contig\_number\_27777SRS019068\_contig\_number\_54157SRS019267\_contig\_number\_contig-100\_2727.33770SRS019397\_contig\_number\_9850SRS022137\_contig\_number\_contig-100\_857.111177SRS024435\_contig\_number\_12781SRS024549\_contig\_number\_4783SRS043001\_contig\_number\_10157SRS043411\_contig\_number\_5104SRS043701\_contig\_number\_contig-100\_219.114862SRS045739\_contig\_number\_7730SRS050422\_contig\_number\_35151SRS050941\_contig\_number\_5966SRS052027\_contig\_number\_14471SRS053335\_contig\_number\_17459SRS054956\_contig\_number\_contig-100\_208.93642SRS056259\_contig\_number\_2075SRS056273\_contig\_number\_31085SRS056519\_contig\_number\_18521SRS058070\_contig\_number\_10582SRS062427\_contig\_number\_contig-100\_7003.46984SRS063040\_contig\_number\_29933SRS063518\_contig\_number\_contig-100\_298.223471SRS075716\_contig\_number\_340SRS076929\_contig\_number\_1927SRS077454\_contig\_number\_5158SRS098644\_contig\_number\_7242SRS098827\_contig\_number\_3032SRS098881\_contig\_number\_contig-100\_377.178952SRS104084\_contig\_number\_13863SRS104485\_contig\_number\_1299SRS104975\_contig\_number\_2438SRS105153\_contig\_number\_36422SRS1055034\_contig\_number\_3362SRS142503\_contig\_number\_49692SRS142781\_contig\_number\_11201SRS143070\_contig\_number\_15722SRS143417\_contig\_number\_6054SRS143598\_contig\_number\_23262SRS143722\_contig\_number\_2745SRS143780\_contig\_number\_16903SRS144362\_contig\_number\_32686SRS144537\_contig\_number\_contig-100\_5260.174104SRS144714\_contig\_number\_18488SRS147919\_contig\_number\_2853SRS148091\_contig\_number\_10214SRS148159\_contig\_number\_43813SRS148424\_contig\_number\_22914SRS148784\_contig\_number\_20712SRS149181\_contig\_number\_22504SRS149879\_contig\_number\_13886SRS893252\_contig\_number\_7579SRS893378\_contig\_number\_20176
species
166486
70


SRS013951\_contig\_number\_9507
species
360807
1
